# From fixing to connecting: parents’ experiences supporting adult children with eating disorders

**DOI:** 10.1186/s40337-024-01140-7

**Published:** 2024-11-23

**Authors:** J. Geller, A. Fernandes, S. Marshall, S. Srikameswaran

**Affiliations:** 1 St. Paul’s Hospital Eating Disorders Program, 1081 Burrard Street, Vancouver, BC V6Z 1Y6 Canada; 2 Department of Psychiatry, University of British Columbia, Vancouver, Canada; 3 Department of Adolescent Health and Medicine, University of British Columbia, Vancouver, Canada

**Keywords:** Parents, Eating disorders, Adult children, Validation, Qualitative, Support

## Abstract

**Background:**

Unlike evidence-based guidelines that exist for families of youth with eating disorders, little is known about the optimal role for families of adult patients. Given issues of patient autonomy and confidentiality, it is common for family members to report high levels of distress, confusion about how to offer support, and feelings of exclusion. Nevertheless, interactions with parents play a critical role in the recovery process. The purpose of this research was to increase understanding of support experiences of parents of adult children while identifying what they believe to be the most beneficial forms of support.

**Methods:**

Sixteen parents of adults who were either recovered or actively engaged in intensive eating disorders treatment participated in semi-structured interviews. Participants were asked to describe the nature of their relationship, beliefs about their child’s support needs, and factors that challenged or facilitated their capacity to offer what they deemed as optimal support.

**Results:**

In the context of receiving support and practicing self-care, parents’ aspiration shifted from fixing the eating disorder to prioritizing a relational goal of unrelenting connection with their child. Achieving this involved three key skills: managing reactions and expectations, learning boundaries, and practicing validation.

**Conclusions:**

This research highlights the ongoing challenges faced by parents, indicating that interventions aimed at supporting their own well-being and fostering connection with their child may be of benefit.

## Introduction

Caring for a child with an eating disorder presents families with an array of emotional, practical, and financial challenges. Parents often fear for their child’s life and struggle to secure access to comprehensive care and support to address medical and psychiatric comorbidities. A recent study reported post-traumatic stress symptoms in 56% of mothers and 62% of fathers whose children were hospitalized for anorexia nervosa [1]. While managing their own reactions to their child’s diagnosis, one of the greatest challenges parents grapple with is determining how to effectively support their child.

The optimal role for parents of youth with an eating disorder is well established in evidence-based treatment. In Family Based Therapy (FBT; [2, 3]) parents of youth are empowered by treatment providers to assume a central role in the functional elements of refeeding their child. This involvement is supported by data demonstrating the benefits of early intervention and by FBT emerging as the leading treatment for youth with eating disorders [4, 5]. When adolescents transition into adult care, where treatment is required to respect patient autonomy and confidentiality, the parental role becomes less clear (e.g., [6]). While research on equipping parents across the developmental spectrum with communication skills and a collaborative stance has received preliminary support (e.g., [7, 8]), little is known about the specific experiences and optimal strategies for parents of adults with eating disorders, particularly those with a longstanding illness.

The knowledge gap of how families of adults can support their children becomes particularly salient in the context of self-compassion, a crucial element in recovery from an eating disorder. Self-compassion has been described as a sensitivity to one’s distress, with the desire to respond in a manner that alleviates suffering [9]. Unlike normalizing eating, which has been shown to benefit from structure and practical support from clinicians and parents of youth, self-compassion involves an internal process linked with both intra and interpersonal relational dynamics [9]. Thus, parents of adults with eating disorders may play an important role in their children developing this skill. Of note, unique barriers to self-compassion have been identified in this population [10], and having these barriers at the beginning of treatment is associated with benefitting less from inpatient and residential care [11]. To date, clinical interventions aimed at enhancing self-compassion have shown promise in this population [12, 13], but the role that family members may play to support this process is not known.

In one study, adults who had recovered from an eating disorder reported that overcoming barriers to self-compassion was associated with receiving validation from care providers [14]. They identified five validating actions: making time and space for me, offering a compassionate perspective, understanding and recognizing my treatment needs, showing me that I can do it, and “walking the runway” (or demonstrating the practice of self-compassion). The actions were linked to key experiences of feeling trust, cared for, empowered, and inspired (see [14]). To our knowledge, these are the first documented recovery-informed recommendations specifically for parents of adults with eating disorders. The extent to which parents are aware of the role validation plays in their child’s eating disorder recovery journey and acquisition of self-compassion, however, remains unexplored.

In sum, while there is consensus regarding the central role that family members play in the recovery of youth with eating disorders, there is limited understanding of the optimal role for family members of adults. Self-compassion has received increased attention in facilitating the recovery journey, and recovered adults with eating disorders suggest that families can help enhance self-compassion by providing validation. The purpose of this research was to increase understanding of parents’ experiences in caring for an adult child with an eating disorder and to explore their beliefs about the most effective ways to support their child’s recovery.

## Methods

### Participants

Parents were recruited through purposeful sampling. Inclusion criteria were to have previously cared for or currently be caring for an adult child with an eating disorder, the child having accessed care at the treatment program where the study took place, and ability to participate in a 90 min interview.

A total of 16 parents participated in the study. Eleven (69%) identified as female, and five (31%) as male. Participants ranged from 49 to 77 years of age (Mean = 65.67). The sample was predominantly White (88%), with one participant of South Asian descent and one of Indigenous descent. Fifteen participants were married and one widowed. Eight were couples (i.e. both parents participated). Most of the sample (81%) were born and raised in Canada. Thirteen (81%) reported their adult child was currently in treatment for an eating disorder. These children ranged from 20 to 45 years of age (Mean = 33.88) and had been living with an eating disorder for 2 to 22 years (Mean = 12.13). All children identified as female (and will be subsequently referred to as daughters). A vast majority (94%) had undergone both outpatient and inpatient treatment, 7 (44%) had participated in day treatment, and all 16 had been in residential treatment. Based on information shared in the interviews, all parents were providing one or more types of support, including financial, pragmatic (e.g., childcare), meal support, and emotional support.

### Procedure

Study information sheets were posted in the waiting area of the treatment program and sent electronically by a family therapist who had established prior contact with parents. Prospective participants were invited to contact the study coordinator to arrange a phone screening to assess eligibility. Informed consent was obtained from all participants prior to the interviews, including communication about confidentiality and the right to withdraw at any time without consequence. All participants were provided with an honorarium.

### Data collection

Semi-structured interviews were conducted by the principal investigator. Interview questions were designed to identify the barriers and facilitators parents experienced while supporting their daughter with an eating disorder (Please see Appendix for list of questions). A visual timeline was collaboratively constructed with participants during the interviews to develop a detailed understanding of key events and their sequence. Interviews were conducted in-person in a private office or through secure videoconferencing software. All interviews were audio-recorded and transcribed verbatim to ensure accuracy in data analysis. Field notes were taken during each interview to capture the interviewer’s observations and reflections. Participants also completed a brief online survey for the purposes of collecting demographic information.

### Reflexivity statement

Two authors (one of whom conducted all interviews) are registered clinical psychologists specializing in tertiary eating disorder treatment and have extensive experience in academic research. Another author is a research assistant with an undergraduate degree in psychology and a substantial background working in tertiary eating disorders programs. Another author is an academic professor with expertise in qualitative analysis and has a longstanding history of collaborations with healthcare researchers, including those in the field of eating disorders. All authors were directly involved in thematic analyses.

### Data analysis

Transcribed interviews were analyzed using a reflexive thematic analysis [15]. This process involved all authors familiarization with the data, generating initial codes, identifying key themes among the codes, rigorous review of the themes, and defining and naming the themes. Through collaborative discussion and a series of meetings to build consensus, all authors contributed to the creation of a model that depicts the associations among the identified themes. The model illustrates the complex emotional and experiential journeys described by parents as they learned and practiced validation skills while navigating the challenges of supporting their adult daughter with an eating disorder.

## Results

**Fig. 1:**
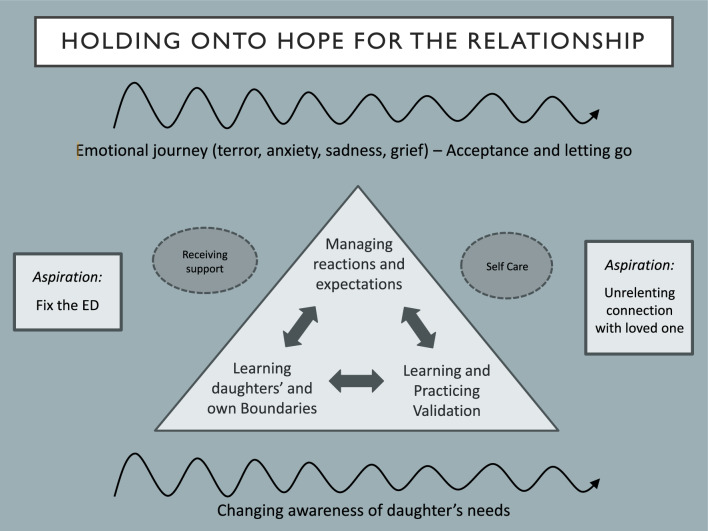
The journey of parents of adult children with eating disorders

Figure 1 offers a model that illustrates the journeys that parents of daughters with eating disorders described navigating in their relationship with themselves, others, and the illness. We conceptualized the parents’ journey as beginning with the aspiration to fix the eating disorder, observing that over time and with experience of treatment, this aspiration evolved into a desire for an unrelenting connection with their daughter. While not every parent articulated the beginning and end point of their journey in precisely these terms, there was nonetheless a collective shift observed across the parent group. This transition involved a departure from solely focusing on resolving the eating disorder, to having the relational goal of cultivating and maintaining the best possible connection with their daughter.

This journey from practical to relational focus was neither straightforward nor linear, and was characterized by an emotional journey, with parents describing their terror, sadness and bewilderment as they adjusted to their daughter changing from someone who they believed they knew well, to someone they at times struggled to understand. Parents also described moving in and out of acceptance and letting go, as they grappled with their own emotions while learning to respond to their daughter’s evolving needs.

Three interconnected skills were fundamental to parents’ journey toward growth and a deeper connection with their daughter. Parents saw the importance of managing their reactions and expectations and recognized that while they were entirely understandable in light of their concerns for their daughter’s health, it could interfere with their ability to respond in a way that served. Learning their daughter’s and their own boundaries facilitated this process. Perhaps most importantly, aspiring for an unrelenting connection with their daughter was linked to the recognition that what she most desired was to feel seen and understood. Parents came to see that their daughter struggled with shame, self-criticism, and confusion over their eating disorder, and many of them described learning that receiving validation was key to supporting both their connection and their daughter’s recovery. This process of offering validation was new and thus for many parents required learning and practicing validation techniques.

Managing their reactions and transforming their relationship with their daughter through the practice of validation did not occur in isolation. Two supportive conditions served as a backdrop for this to take place: receiving support from professionals, friends and family, and practicing self-care. In the next section, each of these model components will be described. Please note that “P” refers to participant.

### Aspiration: fixing the eating disorder

Nearly all parents noted that discovering their daughter had an illness elicited feelings of shock, bewilderment, and fear, and led to an intense drive to fix or get rid of the eating disorder. Parents’ commitment and love was captured by one parent simply stating “you know you’ll take a bullet for them. You’ll do it, and you do it, and you do it.” (P3). For those who were living in the same home as their daughter, the desire to fix the problem was often associated with a period of struggle and conflict around eating.

“There would be yelling matches. Finish your food. Finish what's in your plate. You're not going to move from here till you're done. Get out of the bathroom.” (P5).

Over time, there was disappointment and bewilderment as their efforts were neither positively received nor appreciated.

“We couldn't understand why she couldn't accept our love, couldn't accept our urging her to eat… just be a healthy person. It didn't take long for us to realize, okay, that's not working, and this is way beyond our control.” (P8).

Over time, parents’ yearning to fix was accompanied by a growing awareness that despite having the best of intentions and drive to help, this approach was not effective.

“I'm a fixer. I want to fix. I can fix anything and do anything. I can't fix it. I can't fix her. … I try to accept the situation. It is what it is… I need to fix this. I need to change this, but I can't.” (P4).

Foreshadowing an eventual shift in focus to relational goals, one parent noted: “I think she just wants to be seen, and I think she just wants to be heard. She really wants us to just listen to her. Stop trying to fix things.” (P18).

### Conditions that supported parent journey

On the path to finding a more supportive and fulfilling way of relating to their daughter, parents identified conditions that set the stage for acquiring skills that ultimately better served their relationship with their daughter as well as her recovery. This container included receiving support and self-care.

#### Receiving support

Receiving support was key to decreasing parents’ distress, normalizing their experience, and helping them to feel less isolated. Support occurred in the context of therapy (family, individual, couples), as well as informally from friends, family or professionals.

For many parents, simply learning from a professional that it was acceptable to have conversations about challenging topics gave them permission to address some of the emotional challenges their daughter was grappling with that they had previously believed to be private or off limits.

“We learned, I think through the sessions with [family therapist], that we could—that we didn't die after having difficult conversations… That was new to me to have that kind of intimate talk about feelings and just listen and not try and solve a problem.” (P1).

A subset of parents had the resources to seek out therapy of their own and reported a benefit from individual or couples’ work. Some identified personal challenges of their own that were critical to address in order to support their daughter, such as processing grief or an addiction. For many, attending workshops and meeting other family members with adult children who were further along in their recovery journey was key.

“Hearing people's stories… you're like, oh, my God. That's me. Oh, my God. Look at them. Where are they now? I can be there too. So very helpful.” (P5).

Finally, many parents identified important friends and family who they felt comfortable confiding in. Key experiences were feeling seen and accepted, receiving empathy and compassion, and having an opportunity to discuss their situation with individuals who were psychologically minded. For some, confiding in their spouse was immensely helpful and strengthened their relationship with one another, as well as their capacity to provide ongoing support to their daughter.

#### Self-care

The majority of parents emphasized the importance of tending to their own emotional and physical needs in order to support their evolving relationship with their daughter. In some cases, the motivation for self-care was inspired by a wish to model a healthy way of living for her. Many parents saw self-care as essential to relieving their daughter from the responsibility of caring for them. One mother noted that when she prioritized her well-being, her daughter felt more secure to lean on her for support.

“Her knowing I was getting help helped her as well because it was devastating for her thinking that I was suffering. ‘Mom, I'm so happy you're getting help because I feel better now that I know you're covered as well. I can come to you because I know you go to somebody.’” (P19).

For parents who struggled with mental health or addictions issues of their own, self-care and maintaining sobriety was key to their capacity for learning new skills to support their daughter.

### Parent skill development

The container of receiving support and practicing self-care allowed parents to cultivate three key sets of skills: learning boundaries, managing reactions and expectations, and practicing validation.

#### Learning daughter’s and own boundaries

As parents became aware of their daughter’s changing needs, they increasingly realized that their well-intentioned gestures of love and support needed to adapt accordingly. One mother stated: “my husband and I both, we really just surrendered to the fact that there's—we really need to learn and to watch and to observe and to love and to care in a different way.” (P15).

This awareness was often fueled by recognition that trying to get their daughter to change in accordance with *their* wishes and with *their* timeline was counterproductive, ultimately leading to greater distance in the relationship.

“There was no pushing her to do anything… The boundaries are there. The walls come up. I can see the wall come up and hear the wall come up immediately. It's okay then. Okay, back off. Don't push. Don't push.” (P4).

Common themes that emerged were recognition that their daughters desired more space and privacy, less emotional reactivity, and less or no parental involvement in modifying their eating. One mother simply noted “the one thing she didn't want us—and she made it quite clear—was to poke at her to eat. That worked the other way.” (P19). Recognizing that both space and responsiveness to her daughter’s needs were fundamental, another mother summarized her goal as “to try to stay engaged with her without becoming too enmeshed and without disconnecting.” (P18).

Some parents noted the importance of also clarifying their own boundaries. Examples included one father speaking about not being treated kindly by his daughter or spouse, and a mother choosing to decrease the time she spent providing practical support to her daughter in order to support her own sobriety and mental health.

A second set of fundamental skills was navigating the emotional challenges that arose when parents’ expectations for their daughter diverged with the reality of her life. Parents described experiencing a roller coaster of emotions; anxiety and terror when their daughter’s health was compromised, sadness for her struggles, and grief for the losses that both she and they endured due to the eating disorder. Parents expressed dismay that many of their initial attempts to support their daughter went unappreciated or rejected. While some spoke of times they felt disliked, all described struggling with feelings of frustration and powerlessness as they realized the need to change the way they interacted with their daughter.

“Okay, if I say it this way—it was initially very hard and frustrating to figure that out. It was very frustrating. I used to question myself. I was like, why can't I just be myself with my child? Why do I have to think before I speak? What is this pretend relationship?” (P5).

As illustrated in the model, this emotional journey, characterized by an interplay between acceptance and letting go, was not linear. In some cases, parents heard their daughters asking for space and for parents to manage their expression of distress in her presence.

“… she said something to me like, ‘Yeah, well, look. I know you love me, Mom. But you don't have to love me too much.’ It was like, oh, God, I didn't know you could love too much…When I heard that, I thought, oh, okay. Well, this is going to be hard. I have to love but not love too much. I now think that that was a little bit of code maybe for maybe you were a little overprotective, Mom.” (P9).

Over time, as parents developed better understanding of their daughters’ needs and found new ways of relating, they described being able to interact in a way that the daughter trusted was responsive to her needs and not experienced as overpowering or taking over. This shift was described as accompanied by a sense of relief for both parents and daughters.

#### Learning and practicing validation

Parents’ proficiency at respecting boundaries and managing their reactions and expectations set the stage for developing validation skills.

Many parents described this process as akin to learning a new way of relating to their daughter. As noted earlier, several underscored that it was only after learning that their previous support efforts were not helping that they became open to learning validation skills. Several highlighted that the manner in which they communicated was often more important than what was said.

“Sometimes without realising we are—for us, we're just making a comment or asking a question. But it can be a very accusatory kind of a tone. That is something that I worked on.” (P5).

It is noteworthy that parents in this research had access to a family therapist, and many referenced coaching sessions, in which they had opportunities to prepare and practice prior to having joint sessions with their daughter.

“She [family therapist] would say, ‘She's going to delve into her past. What we need from you in this one is just to listen. If there's a pause, I might look to you and ask you to echo it back. All you can do is echo it back and nod.’ We would do that. We would meet with her [family therapist] later. She would say, ‘Oh, you guys are amazing.’" (P1).

Several parents emphasized the importance of receiving support to not correct their daughter when their perception of events differed, focusing instead on listening and recognizing their daughter’s feelings.

“[Family therapist] helped in hearing some of what maybe her concerns were or whatever. Some of it I felt we were there just to support her, to listen to her, to listen to what she was—what she was saying and what she needed to say to us. In some cases, to hear what she said—again, everybody has a different view of the same picture. Some we felt were not what the situation was in reality. Not being able to counteract that.” (P4).

Most parents indicated that learning validation skills was challenging, requiring effort and regular reminders. Some commented that it was not something that they had learned or experienced in their own families. In several cases, one parent learned the skills more easily than the other and attempted to coach their spouse. One mother summarized her understanding of the benefits of validation as follows:

“Validation helps, really helps because it's—instantly she was like, oh. I am a person. I have feelings. It's being recognised. I'm not just a little loser who's—they're always judging me that I don't do this, I don't do that. I have emotions that are being recognised.” (P5).

### Aspiration: unrelenting connection with daughter

Navigating emotional, practical and financial ups and downs associated with the eating disorder, while learning and adjusting to their daughters’ evolving needs led most parents to the realization that the best way they could support her was through nurturing and sustaining their emotional connection. This realization did not occur in a linear fashion or as a result of a single insight. Rather, it was characterized by a process of accepting that they were not ultimately in control of the eating disorder, and letting go of the notion that certain actions would automatically lead to change or improvement. One mother stated simply "wherever she's going, whatever path she's taking, I want to know who she is." (P15). Parents spoke of coming to a place where they could bear witness to their daughter’s journey with respect, understanding and care, without taking away her determination or autonomy. In the earlier stages, for some, this involved taking time to prepare themselves and their spouses prior to getting together, in order to remain aligned with their aspirations.

“I have been on the opposite side when that wall goes up. I don't want that… That always was my fear. Sometimes with [spouse] it's like, you know what? She's coming over. I don't want any discussion. I'm the boss. I don't want any discussion about anything other than just being present there for the visit.” (P4).

The greatest relief and joy for parents often came when their efforts resulted in positive outcomes, and they felt connected to their daughter following interactions in which there was open sharing about issues of meaning.

### Response to the interviewer

An unexpected observation was made by the interviewer that is worthy of mention. While care was taken to avoid asking leading questions or show preference for a particular type of response, the interviewer nevertheless offered validating feedback to parents’ depictions of the challenges they faced, their efforts at supporting their daughter, and their emotional experiences throughout their journey. Participants appeared to be both relieved and eager for this validating feedback, and for some, it appeared to unlock a floodgate of sharing. Some spoke at length of their frustrations with treatment providers, their efforts to help, and about their own experiences. Others noted that this was the first time they were given an opportunity to be open about their own vulnerability and acknowledge their own needs. Finally, some said that the interview served as a reminder for them to continue having open conversations with their daughter.

## Discussion

Findings from this research suggest that for a parent, supporting an adult child with an eating disorder is an emotional journey, characterized by an evolution in awareness of the child’s needs and their own optimal role in their recovery. Central to this journey was a shift from having a primary motive of curing the eating disorder to a relational aspiration of unrelenting connection. Parents’ narratives were characterized by the complex task of managing their own reactions and expectations, learning and adhering to boundaries, and grappling with how to provide validation when struggling with their own feelings of terror, sadness and grief. This research revealed a story of courage, resilience, acceptance, and letting go. A theme throughout all stories was that amidst the challenges and fluctuations of their daughter’s illness and treatment experiences, parents’ presence and commitment to their daughters endured.

This research was inspired by previous work by our group on recovered individuals, which underscored the positive impact of validation on the acquisition of self-compassion [14]. Parents who participated in this study appeared to have understood this validation message, as their descriptions encompassed all five previously identified levels. Parents’ reported practices most closely resembled the first three levels (making time and space, offering a compassionate perspective, and understanding and matching treatment expectations to their daughter’s readiness). It is noteworthy that parents did not describe providing validation as an easy skill – many reported requiring and benefitting from having a family therapist to assist them in refraining from attempting to influence their daughter’s eating behaviours or offering unsolicited opinions. The level of validation that was least endorsed in this study was “walking the runway” or modelling use of self-compassion in their own lives. This may be due to parents either not actively practicing self-compassion and/or being primarily focused on their daughter’s needs.

Another noteworthy feature of this research was parents’ response to the interviewer. There appeared to be a combination of relief and drive to disclose more in response to having their struggles, efforts, and strengths recognized. Some noted that in the face of their concern about their daughter, they had had few opportunities to tell their own stories. Indeed, it was common for parents to prioritize their daughter’s well-being which overshadowed their own needs. Many participants described benefiting personally from the interview process, with some saying it was a relief to feel heard and understood, and others noting that it was a reminder to continue working on nurturing open, authentic communication with their daughter. It appeared that parents, like their adult daughters, also had a yearning for, and significantly benefitted from, being seen and heard.

This study has several limitations. All participants were recruited from a Canadian tertiary care setting (participated in inpatient and/or residential treatment), and most had children with a longstanding eating disorder (as noted by average illness duration of over 12 years). While it is important to understand this group and learn how best to support them and their children, their experiences may not necessarily reflect those of parents whose adult children have a shorter illness duration and do not require or have access to tertiary care. It is also noteworthy that participants had adult children who had invited them to participate in family therapy, which may not reflect the experiences of families who were not invited or did not have access to family therapy. It is noteworthy that all adult children were daughters, so it is not known whether findings represent the experiences of parents of sons, nonbinary or transgender individuals. Finally, the absence of member checking limited participants’ ability to review and verify the interpretations of their responses. Future research is needed to increase understanding of the experiences of parents from more diverse groups (including ethnicity). In addition, given that adults also have significant connections to partners, future research could explore the experiences of these individuals in supporting adults with eating disorders.

## Conclusion

Findings from this research suggest that parents would benefit from opportunities to speak openly about their experiences in a validating environment, hear the stories of other parents who have navigated and overcome similar challenges, and receive coaching to increase awareness and skills in managing their own and their daughter’s emotional needs. These elements could be included in an intervention specifically tailored for parents of adult children. Future research is needed to develop and test the efficacy of such a treatment at increasing parent and adult child well-being and improving the quality of the parent-adult child relationship.

## Data Availability

To protect the confidentiality of participants as details provided in the interviews could be personally identifying, the data of this study are not publicly available.

## Appendix

### Carer study interview questions

1. How would you describe your relationship with your loved one? How would you describe the impact of the ED on you and your relationship with them? How have you been involved in your loved one’s recovery?
2. Currently, what do you feel your loved one most wishes for you to do to support them? (*prompt practically/emotionally*) What does that look like? What do you think are the reasons that this is what they wish for? Is it easy for you to offer this?

*Describe timeline/recovery journey*

We want to get a sense of your relationship with your loved one in recovery. (Draw a line) Can you tell me about when you first learned about your loved one’s ED – (if might be 2 weeks ago or 5 years). Can you mark off any important events that relate to your relationship with your loved one and any type of support you may have provided.

Draw another line—leave space after so there is still space for imagining the future.

(3) Looking back at the first time point on the journey (use timeline), can you describe what you thought your loved one wanted you to do to support them at that time (practically/emotionally)? (write on timeline). *Note: May need to distinguish between what “ED” wanted vs. “recovery part”.*

- Were you able to offer this support? Is there anything that helped/made it harder?
- Is there anyone in your life (therapist, friends, family) that said/did anything that helped / made it harder for you to offer this support? Describe.
- Was there any impact of people around you? Did your experience any shame or stigma? Anything that helped?
- Switch from change *her* to change *me*?

*(REPEAT 3 for each significant event on the timeline)*

(4) Looking into the future, what do you imagine your loved one will wish from you regarding support? What support do you hope you will offer and what do you imagine will support you or make it harder for that to happen?

## Abbreviations

FBT: Family based therapy
P: Participant
